# Phage-Derived Fully Human Monoclonal Antibody Fragments to Human Vascular Endothelial Growth Factor-C Block Its Interaction with VEGF Receptor-2 and 3

**DOI:** 10.1371/journal.pone.0011941

**Published:** 2010-08-02

**Authors:** Matthias Rinderknecht, Alessandra Villa, Kurt Ballmer-Hofer, Dario Neri, Michael Detmar

**Affiliations:** 1 Institute of Pharmaceutical Sciences, ETH Zurich, Zurich, Switzerland; 2 Philochem AG, ETH Zurich, Zurich, Switzerland; 3 Biomolecular Research, Paul Scherrer Institut, Villigen, Switzerland; University of Southampton, United Kingdom

## Abstract

Vascular endothelial growth factor C (VEGF-C) is a key mediator of lymphangiogenesis, acting via its receptors VEGF-R2 and VEGF-R3. High expression of VEGF-C in tumors correlates with increased lymphatic vessel density, lymphatic vessel invasion, sentinel lymph node metastasis and poor prognosis. Recently, we found that in a chemically induced skin carcinoma model, increased VEGF-C drainage from the tumor enhanced lymphangiogenesis in the sentinel lymph node and facilitated metastatic spread of cancer cells via the lymphatics. Hence, interference with the VEGF-C/VEGF-R3 axis holds promise to block metastatic spread, as recently shown by use of a neutralizing anti-VEGF-R3 antibody and a soluble VEGF-R3 (VEGF-C/D trap). By antibody phage-display, we have developed a human monoclonal antibody fragment (single-chain Fragment variable, scFv) that binds with high specificity and affinity to the fully processed mature form of human VEGF-C. The scFv binds to an epitope on VEGF-C that is important for receptor binding, since binding of the scFv to VEGF-C dose-dependently inhibits the binding of VEGF-C to VEGF-R2 and VEGF-R3 as shown by BIAcore and ELISA analyses. Interestingly, the variable heavy domain (V_H_) of the anti-VEGF-C scFv, which contains a mutation typical for camelid heavy chain-only antibodies, is sufficient for binding VEGF-C. This reduced the size of the potentially VEGF-C-blocking antibody fragment to only 14.6 kDa. Anti-VEGF-C V_H_-based immunoproteins hold promise to block the lymphangiogenic activity of VEGF-C, which would present a significant advance in inhibiting lymphatic-based metastatic spread of certain cancer types.

## Introduction

Lymphangiogenesis is the growth of lymphatic vessels from preexisting ones and the extent of lymphangiogenesis in cancers such as malignant melanoma has been shown to be a predictor of disease progression and survival [1]. The growth of peri- and intratumoral lymphatic vessels, which, in contrary to blood vessels, lack a basement membrane as well as coverage by smooth muscle cells and pericytes and are therefore especially easy to be infiltrated by cancer cells, opens up new ways for metastatic dissemination of the primary tumor. Tumors control the growth of blood and lymphatic vessels in their periphery by the secretion of growth factors. Vascular endothelial growth factor-C (VEGF-C) has been shown to be the main lymphangiogenic growth factor [2], together with VEGF-D [3]. In many tumors, the expression of high levels of VEGF-C has been correlated with lymphatic vessel invasion, the emergence of sentinel and distant lymph node metastasis and overall poor prognosis [4]. Today, tumor metastasis still represents the hallmark of malignancy in cancer.

VEGF-C and VEGF-D exert their action via binding to VEGF-receptors 2 and 3 [2], [3]. While VEGF-R2 is expressed on blood and lymphatic vascular endothelial cells, VEGF-R3 is in the adult expressed normally only lymphatic endothelial cells. Next to their role in metastasis, VEGF-C and -D might also directly activate VEGF-R3 expressed on tumor cells [5], [6], leading to autocrine activation of primary cancer growth and a more aggressive cancer phenotype.

VEGF-C and -D are therefore attractive targets for cancer therapy and agents that are capable of blocking VEGF-C/D and reducing cancer aggressiveness and metastatic dissemination are highly needed to prevent disease progression. Interference with the VEGF-C/D – VEGF-R2/3 system has shown promising results in reducing tumor metastasis and/or primary tumor growth in a number of models. Notably, blocking of VEGF-D by a mouse monoclonal anti-human-VEGF-D antibody [7], [8] was effective in halting primary tumor growth and suppressing local tumor metastasis in a mouse xenograft tumor model. Similarly, neutralizing antibodies against VEGF-R3 inhibited lymph node metastasis [9]–[11] and soluble VEGF-R3, that traps both VEGF-C and VEGF-D, blocked lymphangiogenesis and lymph node metastasis in several models [12], [13].

However, these strategies have potential drawbacks since VEGF-D and VEGF-R3 function in other cells and tissues may also be blocked. VEGF-D is e.g. also expressed in osteoblasts, where it controls bone growth via VEGF-R3 [14]. Blocking of either of these molecules could potentially lead to undesired side effects on bone regeneration.

Blocking of VEGF-C by antibodies has been reported in only a few studies [15]–[18], none of which involved tumor studies. Furthermore, none of these antibodies are of human origin, which hampers their use in human therapy due to immunogenicity. To directly obtain human antibodies, antibody phage-display libraries based on human germline antibody genes offer an alternative route. The fully human ETH-2 Gold antibody phage-display library has been used to isolate binders against a wide spectrum of antigens [19], and antibodies based on binders isolated from the library (e.g. L19, a fully human IgG against the extra domain B of fibronectin, a vascular tumor neo-angiogenesis marker) are currently under clinical development [20].

VEGF-C undergoes excessive processing by proprotein convertases before and after secretion; this processing trims the full length VEGF-C by a N-terminal and C-terminal propeptide and generates ultimately the fully processed, mature ΔNΔC-VEGF-C [21]. This middle third domain contains the VEGF homology domain (VHD), the region of highest homology between VEGF family members and is the most active form of VEGF-C with highest affinity to VEGF-R3, and the only form of VEGF-C that also binds VEGF-R2 [22]. ΔNΔC-VEGF-C therefore represents the most interesting VEGF-C variant to block.

In this study, we used the fully human ETH-2 Gold antibody phage display library to identify antibody fragments specifically binding to the fully processed, mature form of human VEGF-C, ΔNΔC-VEGF-C. We generated a panel of first and affinity matured second-generation single-chain Fragment variable (scFv) binders specifically binding to human ΔNΔC-VEGF-C and were able to show that these scFv block the binding of VEGF-C to both VEGF-R2 and -3. Furthermore, we found that the variable heavy domain (V_H_) of the anti-VEGF-C scFv is sufficient for binding to ΔNΔC-VEGF-C.

## Results

### Selection of anti-VEGF-C scFv

**Figure 1:**
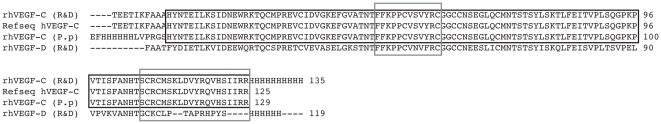
Amino acid sequences of ΔNΔC-VEGF-C and ΔNΔC-VEGF-D variants used in the study. The region of 100% identity within used ΔNΔC-VEGF-C variants is shown within the black frame, the possible epitope regions of VC2.2.2 anti-VEGF-C scFv on ΔNΔC-VEGF-C and the corresponding regions on ΔNΔC-VEGF-D are shown within grey frames. P.p, *P. pastoris*-derived ΔNΔC-VEGF-C; R&D, commercially available mammalian cell-derived ΔNΔC-VEGF-C or ΔNΔC-VEGF-D, respectively.

**Figure 2:**
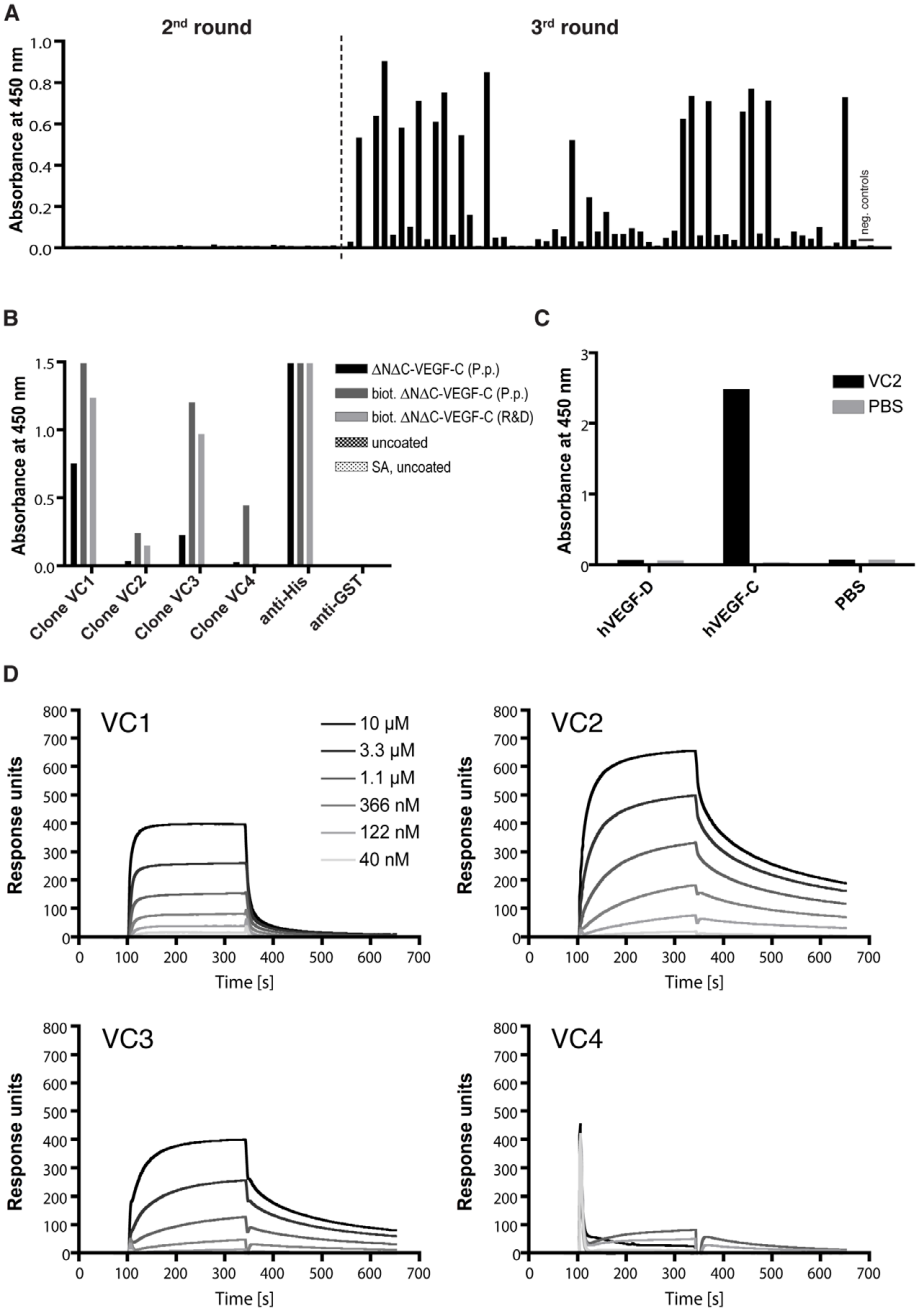
Binding specificities of anti-VEGF-C scFv. (A) ELISA screening of random clones obtained after 2 or 3 rounds of panning against ΔNΔC-VEGF-C. (B) ELISA analysis of representative anti-VEGF-C scFv clones for the 4 different amino acid sequences obtained. Maxisorp or streptavidin-precoated (SA) plates were coated with his-tagged human ΔNΔC-VEGF-C derived from *P. pastoris* or biotinylated his-tagged human ΔNΔC-VEGF-C from mammalian cells or *P. pastoris*, respectively. Control surfaces were left untreated. Antibody fragments and control antibodies were subsequently added and the ELISA was developed as described in Materials and Methods. (C) Cross-reactivity tested by ELISA. Human ΔNΔC-VEGF-C orΔNΔC-VEGF-D (both from mammalian cells) were coated on a maxisorp plate. Anti-VEGF-C scFv clone VC2 or a negative control (PBS only) was added and the ELISA was developed as described in Materials and Methods. (D) BIAcore profiles from the 4 different anti-VEGF-C scFv clones. Different concentrations of protein-A purified scFv were injected on a streptavidin-precoated sensorchip coated with ca. 2000 RU biotinylated mammalian cell-derived ΔNΔC-VEGF-C.

We employed the human fully synthetic ETH-2 Gold antibody phage display library [19] to select for binders against *P. pastoris*-derived human ΔNΔC-VEGF-C (Figure 1). During the course of the selection against VEGF-C, a 974-fold increase in the ratio of output vs. input phage titers, expressed as the enrichment factor, was observed from round 2 to round 3, while the mock selection with uncoated immunotubes yielded a more than 30 times less strong enrichment (Table 1). In the subsequent ELISA screening, 23 of 64 randomly picked clones from round 3 reacted against ΔNΔC-VEGF-C, while none of the randomly picked clones from the 2^nd^ round of selection were positive (Figure 2A). Out of these 23 clones, 4 clones possessing unique amino acid (aa) sequences were retrieved by sequencing. All 4 unique clones were from the lambda subclass; 3 of these 4 clones (VC1, VC2 and VC3) showed 3 identical aa in the randomized complementarity determining region 3 (CDR3) (1 identical aa out of 6 in CDR-L3 (CDR3 of the light chain), 2 identical aa out of 4 in CDR-H3 (CDR3 of the heavy chain)), while the fourth clone (VC4) had a completely unrelated sequence (Table 2).

**Table 1:** Enrichment factors during panning.

Antigen	Round	Input (tu)	Output (tu)	Ratio (out/in)	Enrichment (ratio n/ratio n-1)
VEGF-C	1	5.0×10^12^	1.2×10^6^	2.4×10^−7^	n/a
VEGF-C	2	5.4×10^13^	1.2×10^4^	2.2×10^−10^	0.00093
VEGF-C	3	6.7×10^13^	1.5×10^7^	2.2×10^−7^	974
mock	1	5.0×10^12^	5.0×10^5^	4.0×10^−8^	n/a
mock	2	6.3×10^13^	4.0×10^4^	6.3×10^−10^	0.016
mock	3	4.0×10^13^	8.0×10^5^	2.0×10^−8^	32

Transducing units (tu) before and after panning rounds were determined using titration of transduced colonies. Ratio of output vs. input in the same round and enrichment, i.e. the factor by which the ratio of rescued phages differed from round n-1 to round n was calculated. “Mock” refers to selections with uncoated immunotubes.

**Table 2:** Amino acid sequences of parental and affinity matured anti-VEGF-C scFv.

	CDR-H1			FR2		CDR-H2	CDR-H3						CDR-L1			CDR-L3						
Clone	31	32	33	44	45	64	95	96	97	98	99	100	31a	31b	32	91	92	93	94	95	96	Frequency of recovery
Library	S	Y	A	G	L	K	X	X	X	X	(X)	(X)	S	Y	Y	X	X	X	X	X	X	NA
VC1	S	Y	A	G	**P**	K	**E**	**S**	**S**	**M**	-	-	S	Y	Y	**P**	**I**	**R**	**W**	**A**	**P**	17/64
VC2	S	Y	A	**E**	L	K	**E**	**S**	**L**	**P**	-	-	S	Y	Y	**P**	**R**	**F**	**Y**	**P**	**V**	3/64
VC3	S	Y	A	G	L	**E**	**E**	**S**	**L**	**P**	-	-	S	Y	Y	**P**	**G**	**S**	**E**	**R**	**P**	1/64
VC4	S	Y	A	G	L	K	**W**	**P**	**A**	**T**	**G**	-	S	Y	Y	**V**	**D**	**A**	**W**	**P**	**G**	2/64
VC2.2.2	*Q*	*N*	*Y*	E	L	K	E	S	L	P	-	-	*E*	*N*	*W*	P	R	F	Y	P	V	NA
VC2.2.5	*Q*	*N*	*Y*	E	L	K	E	S	L	P	-	-	*H*	*S*	*Q*	P	R	F	Y	P	V	NA
VC2.1.5	*K*	*N*	*Y*	E	L	K	E	S	L	P	-	-	*K*	*G*	*W*	P	R	F	Y	P	V	NA
VC2.1.20	*Q*	*N*	*Y*	E	L	K	E	S	L	P	-	-	*K*	*N*	*N*	P	R	F	Y	P	V	NA
VC2.2.4	*Q*	*N*	*Y*	E	L	K	E	S	L	P	-	-	*S*	*G*	*N*	P	R	F	Y	P	V	NA
VC2.2.1	*K*	*N*	*A*	E	L	K	E	S	L	P	-	-	*N*	*D*	*Y*	P	R	F	Y	P	V	NA
VC2.2.7	*G*	*N*	*Y*	E	L	K	E	S	L	P	-	-	*K*	*G*	*Y*	P	R	F	Y	P	V	NA
VC2.2.3	*N*	*N*	*Y*	E	L	K	E	S	L	P	-	-	*Q*	*N*	*T*	P	R	F	Y	P	V	NA
VC2.1.6	*S*	*Y*	*A*	E	L	K	E	S	L	P	-	-	*S*	*Y*	*Y*	P	R	F	Y	P	V	NA
VC2.1.26	*N*	*K*	*Y*	E	L	K	E	S	L	P	-	-	*A*	*H*	*M*	P	R	F	Y	P	V	NA
VC2.2.13	*Q*	*S*	*L*	E	L	K	E	S	L	P	-	-	*Q*	*W*	*K*	P	R	F	Y	P	V	NA

Numbering according to Chothia *et al.* [43] VC1 to VC4, first generation anti-VEGF-C scFv; VC2.2.2 to VC2.1.6, positive anti-VEGF-C scFv clones from affinity maturation; VC2.1.26, VC2.2.13, negative scFv clones from affinity maturation; X, random amino acid encoded in the CDR-3s of the ETH-2 Gold library.

As the isoelectric point (pI) of *P. pastoris*-derived ΔNΔC-VEGF-C is 8.3 (as deduced from the amino acid sequence; however, glycosylation also affects the pI), we decided to use 100 mM glycine-HCl pH 2.2 for elution as we speculated that acidic elution is better suited for destabilizing the VEGF-C/phage complex. Interestingly, a parallel panning with 100 mM TEA pH 12 as the elution agent did not yield any binders after 4 rounds of panning.

Since the amino acid sequence of *P. pastoris*-derived ΔNΔC-VEGF-C and of mammalian cell-derived ΔNΔC-VEGF-C differs at the C- and N-terminal ends (Figure 1), all 4 clones were retested on both antigens. The 3 clones with partial identity (VC1, VC2 and VC3) bound to an epitope present on both ΔNΔC-VEGF-C derived from *P. pastoris* and mammalian cells while the fourth, unrelated clone (VC4), bound only to *P. pastoris*-derived ΔNΔC-VEGF-C but not to mammalian cell derived ΔNΔC-VEGF-C (Figure 2B), indicating that the epitopes for the scFvs VC1, VC2 and VC3 are different from the epitope recognized by scFv VC4.

After cleavage of the C- and N-terminal propeptides, both VEGF-C and VEGF-D exist as the fully processed, mature ΔNΔC protein. This central VEGF homology domain (VHD) in VEGF-C and VEGF-D is highly homologous (more than 50% identity and more than 70% homology, Figure 1). However, clone VC2 did not bind to mammalian cell-derived ΔNΔC-VEGF-D, confirming the specificity for VEGF-C (Figure 2C).

All 4 clones were next analyzed with surface plasmon resonance (SPR) on a streptavidin coated BIAcore chip to which biotinylated mammalian cell-derived ΔNΔC-VEGF-C was immobilized. This analysis revealed different binding profiles, and clone VC2 was chosen to be used for affinity maturation since it displayed the best apparent binding affinity for VEGF-C (Figure 2D).

### Affinity maturation

Antibody phage libraries to be used in affinity maturation selections were constructed essentially as described [23], based on the amino acid sequence of clone VC2. Randomizations were engineered in CDR1 of both heavy and light chains. The obtained library consisted of 3.3×10^6^ clones, of which 14 randomly picked clones all contained the scFv insert as assessed by colony PCR analysis. More than 90% of 96 randomly picked clones were capable of expressing a soluble scFv antibody fragment, as assessed by Dot-Blot analysis (data not shown). Sequencing of 6 random clones revealed completely randomized aa sequences in the desired CDR-H1 and CDR-L1, while the VC2 parental aa sequence was retained in all other residues (data not shown).

**Figure 3:**
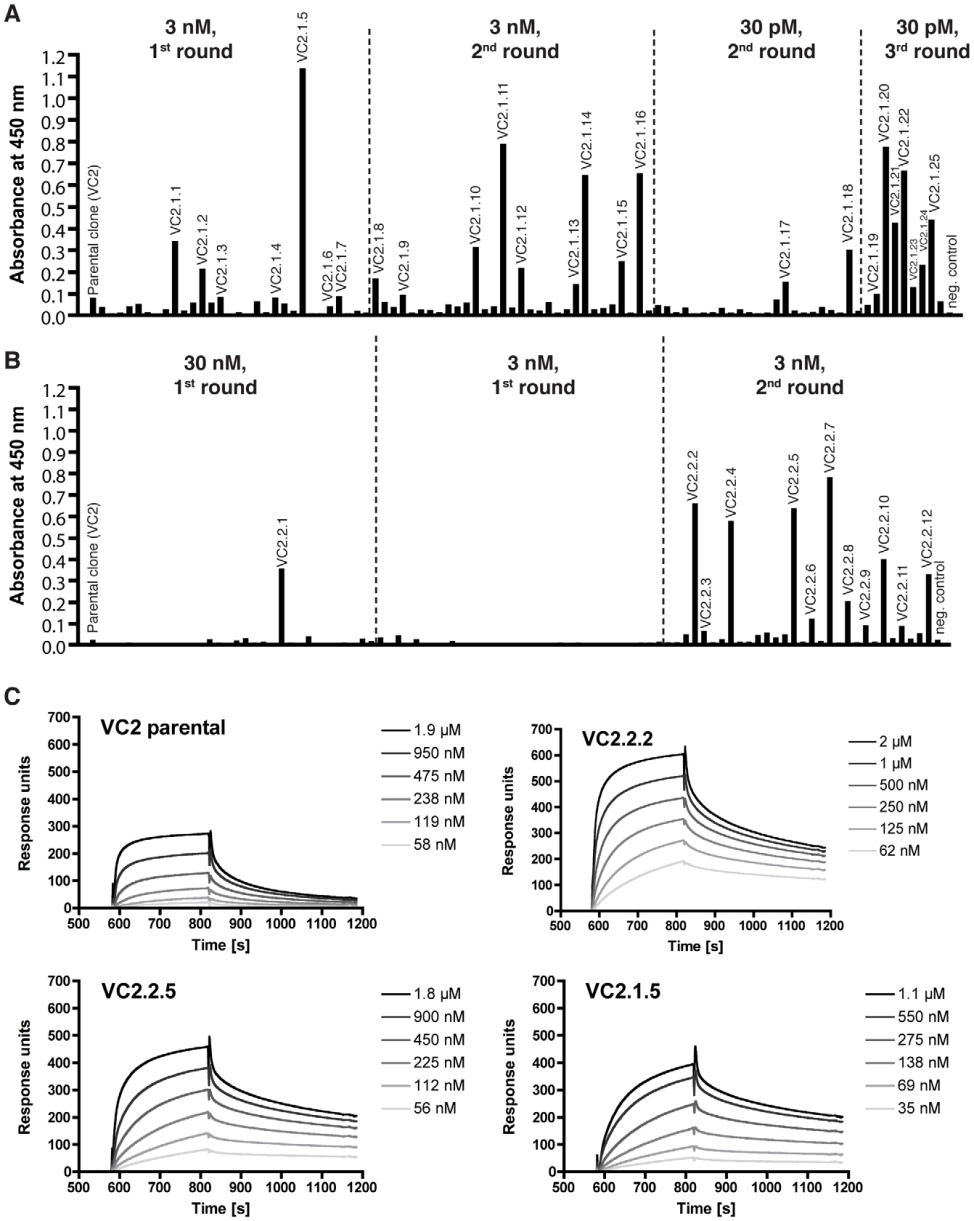
Affinity matured anti-VEGF-C scFvs possess a higher affinity. (A, B) ELISA analysis of bacterial supernatant from randomly picked affinity matured clones after 1 to 3 rounds of selection on biotinylated (A) *P. pastoris*-derived or (B) mammalian cell-derived ΔNΔC-VEGF-C. (C) BIAcore profiles of monomeric affinity matured anti-VEGF-C scFvs. Monomeric fractions of protein-A purified scFv were prepared by FPLC and injected as 2-fold dilution series on a streptavidin-sensorchip coated with 2000 RU biotinylated ΔNΔC-VEGF-C derived from mammalian cells.

After 1 to 3 rounds of panning of this affinity maturation library on varying concentrations of biotinylated *P. pastoris* and mammalian cell-derived ΔNΔC-VEGF-C, random clones from several selections were analyzed by ELISA. This analysis showed that more selection rounds and higher antigen concentration increased the percentage and signal intensity of binders identified (Figure 3A and 3B).

Sequencing of 10 positive clones that showed some of the highest ELISA signals and of 2 negative controls was subsequently performed. One clone (VC2.1.6) was identified to be identical to the parental scFv VC2, and clones VC2.2.5 and VC2.1.11 were identical to each other. The positive clones showed a converging selection of CDR-H1 and CDR-L1 sequences (Table 2). In CDR-H1 sequences, X-Asn-Tyr (X-N-Y) was selected in 7 of 8 cases, where X was always a hydrophilic residue (Asp, Glu, Asn, Gln, His, Lys, Arg; D, E, N, Q, H, K, R) or a glycine. In CDR-L1, sequences were more heterogeneous. CDR1s of negative controls exhibited a more random pattern. An amber stop codon (TAG, coding for Stop or Gln) was found in the CDR-H1 of 4 out of 8 positive clones and in the CDR-L1 of 1 out of 8 positive clones and was corrected to CAG by PCR.

Three clones (VC2.2.2, VC2.2.5 and VC2.1.5) were chosen for further characterization, since they showed the strongest ELISA signal. Protein-A purified fractions were further purified by size-exclusion chromography and monomeric preparations were used for BIAcore analysis. Dissociation constants of 22, 35 and 43 nM, respectively, were measured in this analysis, an improvement by almost 4-fold compared to the parental scFv VC2 (81 nM) (Figure 3C and Table 3). VC2.2.2, which exhibited the lowest K_d_, was subsequently used for further analysis of blocking capacity.

**Table 3:** Comparison of the kinetic constants of the affinity matured anti-VEGF-C scFvs.

scFv	k_on_ (1/Ms)	k_off_ (1/s)	K_d_ (nM)	K_d_ improvement relative to VC2
VC2	4.45×10^4^	3.58×10^−3^	81	-
VC2.1.5	2.39×10^4^	1.02×10^−3^	43	1.9
VC2.2.2	5.65×10^4^	1.22×10^−3^	22	3.7
VC2.2.5	2.85×10^4^	1.00×10^−3^	35	2.3

The kinetic constants were fitted from dilution series of monomeric scFv preparations with BIAevaluation3.1 software using a 1∶1 Langmuir binding model.

### VC2.2.2 anti-VEGF-C scFv blocks binding of VEGF-C to VEGF-R2 and VEGF-R3

#### BIAcore assay

Fully processed human VEGF-C (ΔNΔC-VEGF-C) exerts its action via binding to VEGF-R2 and VEGF-R3 and binds the two receptors with affinities of 410 and 135 pM, respectively [22]. Fusions of VEGF-R2 or VEGR-R3 with the crystallizable fragment (Fc) of human IgG (VEGF-R2-Fc or VEGF-R3-Fc) were bound to an anti-Fc coated BIAcore chip to generate a homogenous receptor surface. ΔNΔC-VEGF-C was then passed over the receptor surface and binding occurred. In both cases, the receptor surface was able to bind almost equimolar amounts of VEGF-C (data not shown). To assess the neutralizing capacity of VC2.2.2 scFv, ΔNΔC-VEGF-C was preincubated with different concentrations of VC2.2.2 scFv to allow for the formation of the scFv-antigen complex, and this complex was then injected on the receptor surface.

**Figure 4:**
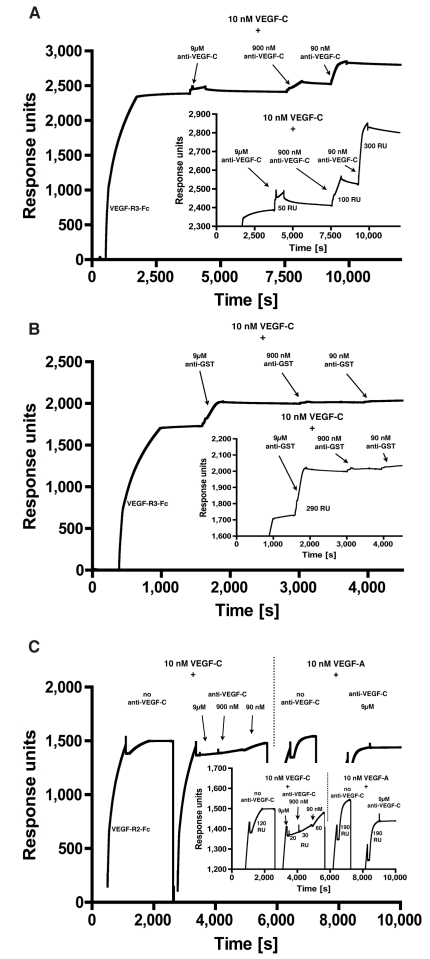
VC2.2.2 blocks binding of VEGF-C to VEGF-R2 and VEGF-R3 as measured by SPR. VEGF-R3-Fc was bound to a CM5-sensorchip coated with anti-human IgG antibody. 10 nM ΔNΔC-VEGF-C was then preincubated with a 9 to 900 times molar excess of (A) anti-VEGF-C scFv or (B) control scFv and injected on the VEGF-R3-Fc surface and the amount of binding of ΔNΔC-VEGF-C was measured by SPR. (C) VEGF-R2-Fc was bound to a CM5 sensorchip coated with anti-human IgG antibody. 10 nM of ΔNΔC-VEGF-C or VEGF-A with or without preincubation together with anti-VEGF-C scFv were then injected on the VEGF-R2 surface and bound VEGF-A or VEGF-C was measured by SPR.

The binding of ΔNΔC-VEGF-C to immobilized VEGF-R3 was dose-dependently inhibited by anti-VEGF-C scFv VC2.2.2 (Figure 4A), but not by the irrelevant control scFv directed against glutathione-S-transferase (GST) (Figure 4B). With 900-fold molar excess of VC2.2.2 scFv, an 86% reduction of response was achieved.

On the VEGF-R2 coated surface, binding of ΔNΔC-VEGF-C was also dose-dependently inhibited by injection of a mixture of ΔNΔC-VEGF-C preincubated for 30 minutes with anti-VEGF-C scFv VC2.2.2 (Figure 4C). With 900-fold molar excess of VC2.2.2 scFv, an 81% reduction of response was achieved. Binding of 10 nM VEGF-A to VEGF-R2 resulted in a response comparable to the binding of 10 nM ΔNΔC-VEGF-C but was not blocked by a 900-fold molar excess of anti-VEGF-C scFv VC2.2.2, demonstrating the specificity of the anti-VEGF-C scFv (Figure 4C).

#### Competitive ELISA

The flow-based BIAcore VEGF-C neutralization assay measures the neutralizing potency of the VC2.2.2 anti-VEGF-C scFv only during a short time span, since the scFv-bound VEGF-C had only a short lasting possibility (the passage through the flow-cell requires seconds) to dissociate from the scFv and to bind to the immobilized VEGF-R on the chip. The equilibrium between dissociation from the scFv and association to the VEGF-R might not be reached during the passage through the flow cell. Therefore, we next used a competitive ELISA to characterize the neutralization potency of the anti-VEGF-C scFv over a longer time span.

**Figure 5:**
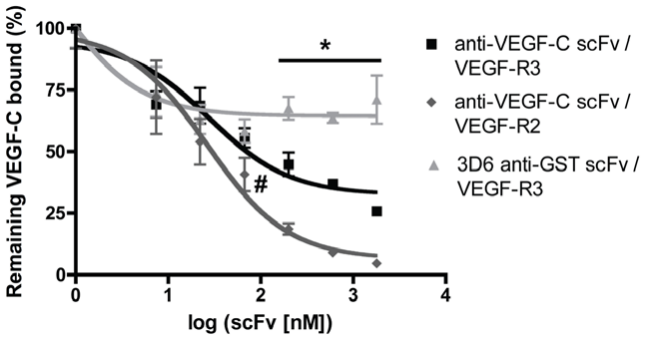
VC2.2.2 blocks binding of VEGF-C to VEGF-R2 and VEGF-R3 as measured by competitive ELISA. 2 nM biotinylated ΔNΔC-VEGF-C was preincubated with varying amounts of anti-VEGF-C scFv or control scFv and added on a VEGF-R2 or VEGF-R3 coated microtiter plate. Plotted datapoints are means from 4 replicates ± SEM. The datapoints were fitted to a sigmoidal dose-response curve model using GraphPad Prism 4. Inhibition of VEGF-C binding by anti-VEGF-C scFv reached significance vs the control scFv (*, p<0.05, Student's t-test) at molar excess of 100× more anti-VEGF-C scFv vs VEGF-C. #: p = 0.067 for anti-VEGF-C/VEGF-R2 vs control at molar excess of 33× more anti-VEGF-C than VEGF-C.

We found that VC2.2.2 anti-VEGF-C scFv dose-dependently blocked the binding of biotinylated ΔNΔC-VEGF-C to immobilized VEGF-R, while the anti-GST control scFv 3D6 did not block this binding (Figure 5). Blocking of VEGF-R2 binding was more efficient than blockage of VEGF-R3 binding, in agreement with the findings in the BIAcore assay and the fact that affinity of ΔNΔC-VEGF-C to VEGF-R3 is higher than to VEGF-R2 [22]. With a 900-fold molar excess of VC2.2.2 anti-VEGF-C scFv, binding of biotinylated ΔNΔC-VEGF-C to VEGF-R2 was inhibited by 95%±1.0%, while the binding to VEGF-R3 was blocked by 74%±3.5%. Blocking reached a significance level of p<0.05 (Student's *t*-test) vs. 3D6 irrelevant control antibody for VC2.2.2 anti-VEGF-C scFv concentrations ≥200 nM or 100-fold molar excess over biotinylated VEGF-C.

### VC2.2.2 anti-VEGF-C scFv binds to an epitope on ΔNΔC-VEGF-C implicated in VEGF receptor binding

To locate the epitope on ΔNΔC-VEGF-C to which VC2.2.2 anti-VEGF-C scFv binds, a peptide microarray consisting of overlapping 15-mer peptides spanning the whole ΔNΔC-VEGF-C aa sequence was used. Cy3-labelled VC2.2.2 scFv and Cy5-labelled 3D6 scFv were allowed to competitively bind to the peptide array. Upon scanning, the ratio of median signals from Cy3 vs Cy5 channels were used to generate a list of peptides bound by the respective scFvs.

*P. pastoris*-derived ΔNΔC-VEGF-C, the positive control protein, emerged as the top-hit from this scan, being bound more than 100 times stronger by the anti-VEGF-C scFv than by the irrelevant scFv (Table 4). This validates the usefulness of the peptide-array. Target peptides more strongly bound by the VC2.2.2 anti-VEGF-C scFv vs. the irrelevant scFv 3D6 contained the sequence FFKPPCVSVYRC (bound more than 22 times stronger by VC2.2.2 than irrelevant control) as well as the C-terminal sequence spanning SCRCMS to RQVHSIIRRHHHH (bound more than 4 times stronger).

**Table 4:** Epitope mapping.

Peptide/Protein	log2 ratio	Stdev	αVEGF-C Signal	Stdev	αVEGF-C SNR	Stdev	αGST Signal	Stdev	αGST SNR	Stdev
ΔNΔC-VEGF-C	**6.26**	0.19	**8346**	5844	**11.4**	5.0	**202**	79	**2.9**	1.1
ΔNΔC-VEGF-C	**6.18**	0.12	**8531**	5797	**25.8**	30.0	**213**	78	**3.5**	2.1
TNTFFKPPCVSVYRC	**5.98**	0.03	**51361**	1256	**112.7**	9.5	**917**	48	**16.9**	5.5
FFKPPCVSVYRCGGC	**4.50**	0.12	**45671**	391	**120.2**	89.2	**2114**	184	**46.4**	17.6
SCRCMSKLDVYRQVH	**4.29**	0.17	**34911**	1322	**332.6**	215.7	**1872**	142	**49.4**	12.4
RQVHSIIRRHHHHHH	**4.22**	0.17	**22414**	1071	**166.6**	43.8	**1302**	86	**34.8**	6.4
VYRQVHSIIRRHHHH	**3.69**	0.03	**32602**	1576	**63.8**	29.1	**2615**	189	**26.4**	3.1
KLDVYRQVHSIIRRH	**2.67**	0.07	**25280**	2821	**123.0**	68.9	**4041**	269	**64.3**	14.5
PPCVSVYRCGGCCNS	**2.13**	0.05	**1555**	65	**25.0**	2.4	**415**	37	**9.0**	2.1
SFANHTSCRCMSKLD	**1.58**	0.12	**1248**	54	**16.1**	4.5	**459**	9	**6.8**	3.8
NHTSCRCMSKLDVYR	**1.53**	0.24	**7390**	1362	**170.9**	95.4	**2577**	126	**58.3**	26.9
VTISFANHTSCRCMS	**1.24**	0.11	**2945**	165	**65.6**	24.5	**1272**	18	**31.9**	3.7
human IgG	**0.07**	0.01	**9348**	531	**41.3**	7.5	**8917**	583	**31.3**	4.8
human IgG	**0.03**	0.06	**9071**	491	**34.0**	15.0	**8833**	423	**27.3**	9.3
mouse IgG	**−0.05**	0.04	**4873**	474	**24.4**	11.2	**4969**	363	**27.1**	8.6
mouse IgG	**−0.05**	0.01	**4885**	268	**43.0**	6.9	**4994**	310	**32.1**	5.5
CMSKLDVYRQVHSII	**−0.36**	0.13	**401**	43	**7.0**	1.1	**388**	32	**4.5**	3.0
QCMNTSTSYLSKTLF	**−0.96**	0.16	**1183**	81	**15.8**	5.8	**2065**	121	**10.4**	1.2

Values are arithmetic means and standard deviations from 3 subarrays. SNR: signal to noise ratio, log2 ratio are log_2_ (VC2.2.2 anti-VEGF-C/3D6 anti-GST).

All peptides that generated data with errors, SNRs <2 in both channels and signal intensities ≤100 in both channels were filtered out.

Shown is a representative array from at least 3 replicates. Dye-swap arrays yielded similar results.

**Figure 6:**
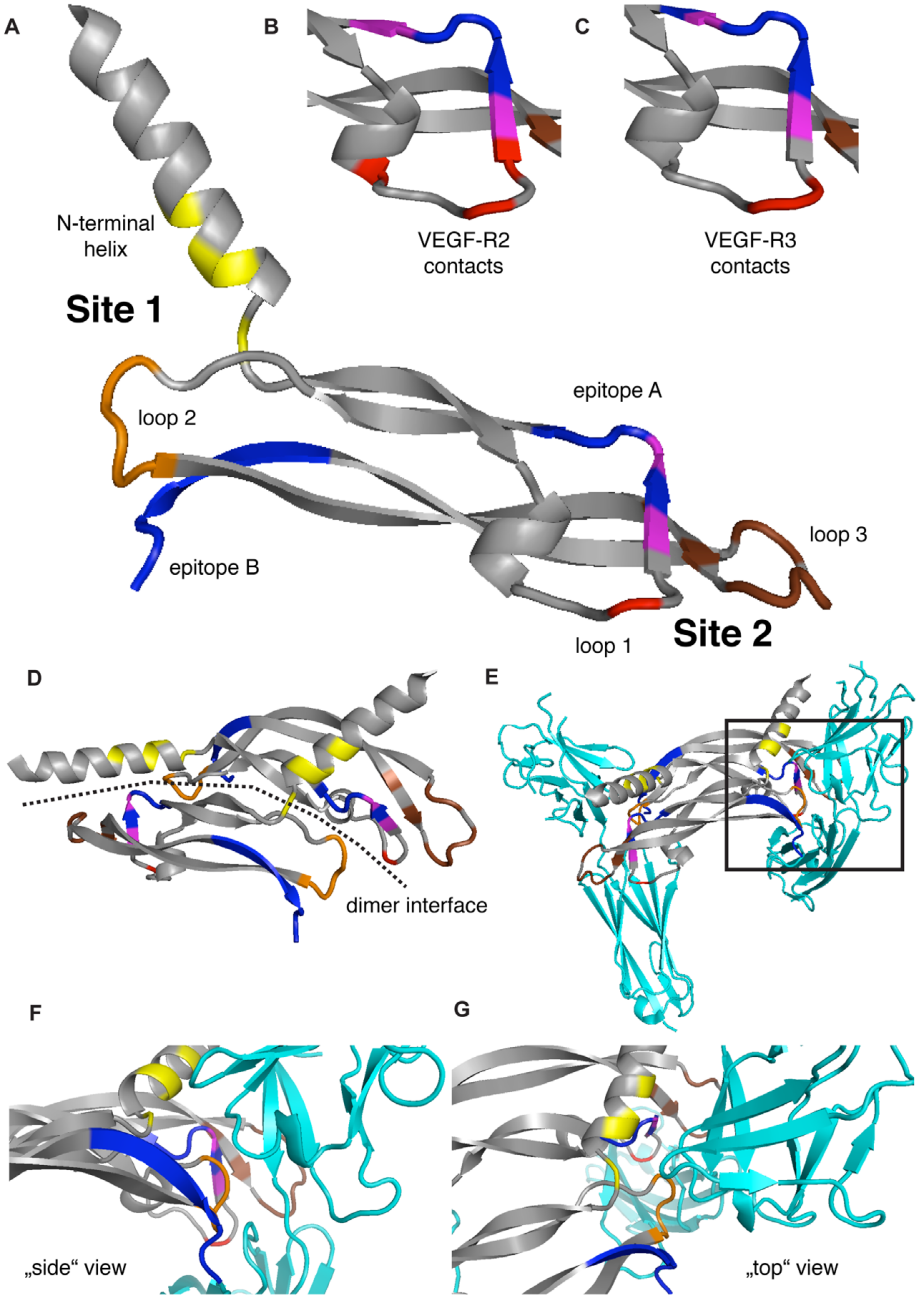
Possible VC2.2.2 anti-ΔNΔC-VEGF-C epitope-localization within or near the receptor-binding region on ΔNΔC-VEGF-C. (A) The VEGF-C residues contacting VEGF-R2 as reported in [32] are represented in yellow (N-terminal helix), red (loop 1), orange (loop 2) and brown (loop 3). The two epitope stretches identified in the peptide scan are colored in blue. From epitope B, only SCRCMSKL is shown, the C-terminal end is missing in the reported structure. Overlaps of epitope A (FFKPPCVSVYRC) and receptor-contacting residues in loop 1 are colored in purple. Residues in loop 1 found to affect (B) VEGF-R2-binding or (C) VEGF-R3-binding by mutational analysis [24] are shown with the same colors as in (A). The localization of the epitopes within the VEGF-C dimer is shown in (D) and their interference with the boxed interface of VEGF-R2 (cyan) is shown in (E), with magnifications in “side” view (F) and “top view” (G). The pdb file 2X1X was used for the representation (www.pdb.org).

Interestingly, the FFKPPCVSVYRC sequence maps to the region on VEGF-C that is most important for receptor binding to VEGF-R2 and VEGF-R3 (Figure 6) [24] and contains Cys156, the key-residue responsible for VEGF-R2 binding [25]. Importantly, ΔNΔC-VEGF-D, which is not bound by VC2.2.2, features two different residues in the FFKPPCVSVYRC region, which might be responsible for the loss of binding (Figure 1). The C-terminal sequence SCRCMS to RQVHSIIRRHHHHHH lies in proximity to the loop 2 in the site 1 receptor binding interface of VEGF-C (Figure 6) and binding of anti-VEGF-C to this region could therefore also sterically hinder the binding of VEGF-C to its receptors.

### The variable heavy domain V_H_ of VC2.2.2 is sufficient for binding to VEGF-C

**Figure 7:**
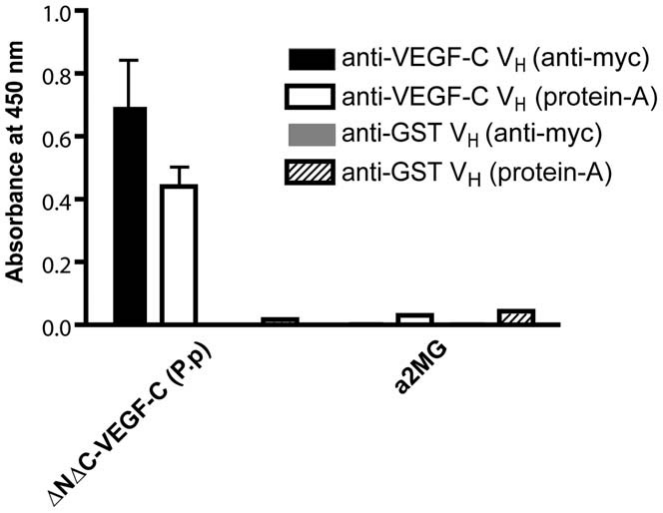
The VC2.2.2 V_H_ is sufficient to bind ΔNΔC-VEGF-C. Binding of the single V_H_-domain of VC2.2.2 to ΔNΔC-VEGF-C and an unrelated antigen (alpha-2-macroglobulin, a2MG) was tested by ELISA using either anti-myc and anti-mouse HRP or protein-A-HRP as detection compounds.

Bacterial supernatant from IPTG induced cultures expressing VC2.2.2 V_H_-myc or a control V_H_-myc were checked by ELISA for reactivity against ΔNΔC-VEGF-C. Binding to ΔNΔC-VEGF-C could be observed, while no unspecific stickyness to alpha-2-macroglobulin was seen (Figure 7). Detection with protein-A was also successful, pointing to a generally correct folding of the V_H_, since protein-A binds to a conformational epitope on the opposite face of the former dimerization interface between V_H_ and the variable light domain V_L_ [26].

### Anti-VEGF-C scFvs contain hydrophilic camelid V_H_H-like mutations in the V_H_:V_L_ dimerization interface

Upon reexamination of the anti-VEGF-C scFv, mutations in the framework region 2 (FR2) and adjacent regions of the heavy chain could be identified. In VC2 and its daughter clone VC2.2.2, residue 44 was mutated from glycine (as encoded in the ETH-2 Gold library) to glutamic acid (Table 2), by a purinic single nucleotide mutation from GGG to GAG, making it more hydrophilic. The FR2 region lies within the dimerization interface of the V_H_ and V_L_ domains and loss of the V_L_ domain leads to resurfacing of hydrophobic residues within the former dimerization interface and could lead to decreased solubility and enhanced aggregation. The G44E substitution and other hydrophilic substitutions in the FR2 region are therefore a hallmark of the variable heavy domain V_H_H in camelid heavy chain antibodies, naturally occurring immunoproteins devoid of light chains [27], [28]. VC1 contains a mutation in the FR2 region where the hydrophobic leucine at position 45 is replaced by the less hydrophobic proline, caused by a CTG to CCG transition mutation (Table 2). L45 is also one of the typical residues altered in camelid V_H_H, although the canonical residue there is the hydrophilic arginine. However, the CGG arginine codon can only be reached from the CTG proline codon by a T to G pyrimidine/purine transversion, but transversions are about an order of magnitude less frequent than purinic (A/G) or pyrimidinic (C/T) transitions [29], [30]. For a single pyrimidinic transition, proline is the most hydrophilic residue “reachable” from the leucine codon CTG. VC3 contains a mutation in C-terminal end of CDR-2, where the already hydrophilic lysine at position 64 is substituted by the similarly hydrophilic glutamic acid, by a AAG to GAG transition mutation (Table 2). VC4, which binds only to *P. pastoris*-derived ΔNΔC-VEGF-C but not to mammalian cell-derived ΔNΔC-VEGF-C does (outside CDR-3s) not deviate from the amino acid sequence encoded in the library (Table 2).

### Anti-VEGF-C scFv with camelid-like mutations show unfavorable gel-filtration profiles, but the single V_H_ is partly stabilized

**Figure 8:**
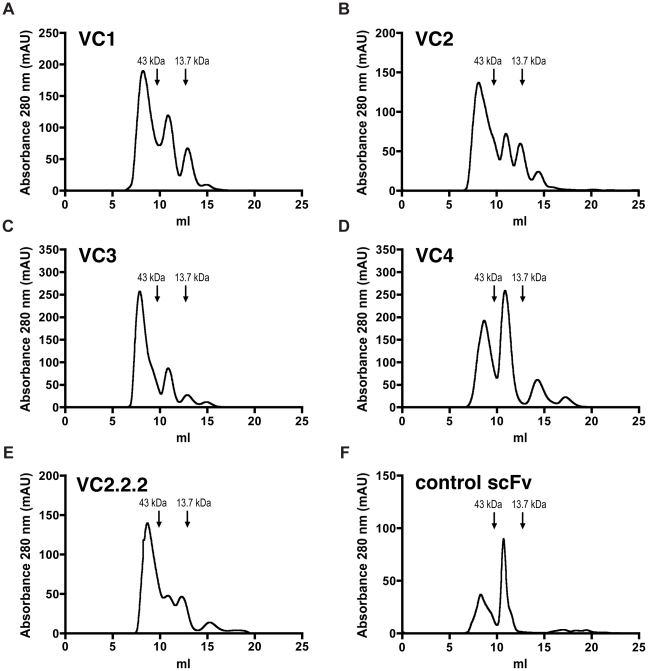
SEC gel-filtration profiles of anti-VEGF-C scFvs. Protein-A purified scFv were injected on a Superdex 75 10/300 GL size-exclusion gel-filtration column. Markers represent the major elution peaks of the molecular mass standards ovalbumin (43 kDa) and ribonuclease A (13.7 kDa).

When analyzed on a Superdex 75 size-exclusion gel-filtration column, the anti-VEGF-C scFvs VC1, VC2 and VC3 as well as the VC2 daughter VC2.2.2 eluted with several peaks indicating presence of dimers, multimers or higher aggregates, and the monomeric peak at about 11 ml was markedly reduced when compared to a control scFv. VC4, which does not contain any deviation from the ETH-2 Gold library sequence but does only bind to *P. pastoris*-derived ΔNΔC-VEGF-C, showed a more favorable elution profile. Several peaks with later elution time-points were also present both in VC1 to VC3 and VC4, indicating probable proteolytic products (Figure 8A–F).

**Figure 9:**
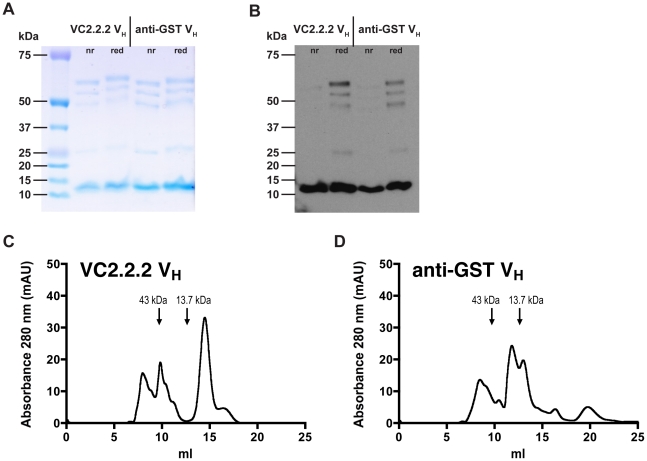
SDS-PAGE and immunoblot analysis as well as SEC gel-filtration profiles of anti-VEGF-C VC2.2.2 V_H_ and anti-GST V_H_. Protein-A purified V_H_ under non-reducing (nr) and reducing (red) conditions were separated by SDS-PAGE and (A) stained using Coomassie-Blue or (B) immunoblotted using an anti-myc antibody followed by anti-mouse HRP. Protein-A purified (C) anti-VEGF-C VC2.2.2 V_H_ or (D) anti-GST V_H_ were injected on a Superdex 75 10/300 GL size-exclusion gel-filtration column. Markers represent the major elution peaks of the molecular mass standards ovalbumin (43 kDa) and ribonuclease A (13.7 kDa).

As determined by SDS-PAGE and anti-myc immunoblot, protein-A purified myc-tagged VC2.2.2 V_H_ and anti-GST V_H_ both run at about 12 kDa (Figure 9A,B), although their molecular masses are calculated from their primary amino acid sequences as 14.6 kDa. When subjected to size-exclusion gel-filtration, the VC2.2.2 V_H_ showed a major peak corresponding to the putative V_H_ monomer [31], eluting just after the 13.7 kDa marker (Figure 9C), while the non-“camelized” anti-GST V_H_ showed peaks corresponding to higher molecular weights (Figure 9D).

## Discussion

In this study, we describe the development of function-blocking monoclonal antibody fragments against human ΔNΔC-VEGF-C. The blocking capabilities were confirmed at the molecular level using BIAcore and competitive ELISA. Antibody phage-display was used to select 4 lead binders from the ETH-2 Gold antibody phage display library. From these 4 binders, one was specific for an epitope only present on *P. pastoris*-derived ΔNΔC-VEGF-C, which was used for selection, while the other 3 clones bound to an epitope in the conserved region of ΔNΔC-VEGF-C. Binding to the His-tag could be excluded, since ΔNΔC-VEGF-D, to which the scFv are not binding, also contains a His-tag.

Via affinity maturation of clone VC2, daughter clones with up to 4-fold improved affinity (22 nM, clone VC2.2.2) were obtained. The affinities of these daughter clones are in the same range as described for VD1, a function-blocking mouse IgG against human ΔNΔC-VEGF-D [8].

The blocking of both VEGF-C binding to VEGF-R2 and VEGF-R3 indicates that the epitope for VC2.2.2 scFv on ΔNΔC-VEGF-C might be localized in a region that is important for binding to both of the receptors. Indeed, data generated by the epitope-mapping peptide array hint to a specific binding to the VEGF receptor binding domain on ΔNΔC-VEGF-C. Recently, the three-dimensional crystal structure of ΔNΔC-VEGF-C in complex with one of its receptors, VEGF-R2, was solved by X-ray diffraction [32]. The receptor binding domains of VEGF-C were mapped to two sites consisting of three loops and part of the N-terminal helix on VEGF-C (Figure 4A). Site 1 is composed of the N-terminal helix alpha1 (His113 – Thr129) and the loop L2 (Asn167 – Leu171). Site 2 consists of the loops L1 (Asp139 – Pro155) and L3 (Ile188 – Pro196). Site 1 is provided by one VEGF-C monomer, while site 2 comes from the antiparallel second VEGF-C monomer [32] (Figure 4B). The receptor binding domains to both VEGF-R2 and VEGF-R3 have also earlier been analyzed by mutational analysis of VEGF-C and these data correlate well with the structural results for VEGF-R2 [24]. The VEGF-C sites and residues responsible for binding to VEGF-R3 are essentially the same as those making contact with VEGF-R2.

F_151_FKPPCVSVYRC_162_, the top-hit peptide from the epitope scan, maps to loop L1 in site 2 and the presumed dimerization interface of ΔNΔC-VEGF-C. Loop L3 in site 2 and, when dimerized, loop L2 and helix alpha1, that contain other important residues for VEGF receptor binding are located in the spatial vicinity of the putative epitope. Binding of VC2.2.2 anti-VEGF-C scFv could therefore possibly interfere with dimerization of VEGF-C and sterically hinder VEGF-C loops 1 and 3 from making contacts to the receptor (Figure 6). Importantly, ΔNΔC-VEGF-D, which is not bound by VC2.2.2 scFv, features two different residues in the FFKPPCVSVYRC region, which might be responsible for the lack of cross-reactivity towards VEGF-D (Figure 1).

The observation that the V_H_ of VC2.2.2 is sufficient for binding to VEGF-C is intriguing. It might explain why during affinity maturation in the first round, similar advantageous sequences were selected in CDR-H1, while in CDR-L1 no consensus could be observed. The identification of mutations in all three scFv binding to mammalian cell-derived ΔNΔC-VEGF-C offers a possible explanation for these observations. Transitional single nucleotide mutations leading to the hydrophilic FR2 substitutions G44E in VC2 (and its daughter clone VC2.2.2) and L45P in VC1 as well as the CDR2 substitution K64E in VC3 obviously lead to a selection advantage during the panning against ΔNΔC-VEGF-C. It can be speculated that ΔNΔC-VEGF-C favors binding of small single V_H_ domains in contrast to paired Fvs, maybe due to the heavy glycosylation that may obstruct access to proteinaceous epitopes. Hints that V_L_ and V_H_ are not paired correctly in the scFvs come from gel-filtration profiles of first and second generation anti-VEGF-C scFvs. The first generation scFv VC1, VC2 and VC3, that contain mutations and bind to mammalian cell-derived ΔNΔC-VEGF-C as well as the VC2 daughter clones all showed unfavorable gel-filtration profiles with an enhanced ratio of scFv multimers or aggregates vs. monomers, while the first generation scFv VC4, that does not contain mutations and binds only to *P. pastoris*-derived ΔNΔC-VEGF-C, shows a more favorable gel-filtration profile with the monomer as the most prominent peak (Figure 8). The mutations could be the cause of the special binding properties or an evolutionary adaptation of the scFv-coding gene towards higher solubility of the expressed non-properly folded scFv. Both would result in a selection advantage, either during panning of the scFv-bearing phage or during growth of the phagemid-bearing *E.coli.* Interestingly, camelid V_H_Hs have been reported to often bind to unusual and hidden protein epitopes, e.g. clefts in enzymes. This has been attributed to their especially long CDR3 loop, which protrudes and is able to insert itself into concave epitopes [33]–[35]. In contrast, normal Fv paratopes are flat or contain a cleft themselves and normally bind to flat or convex epitopes. Interestingly, the FFKPPCSVYRC epitope candidate emerging from the peptide scan lies partly buried in the dimerization interface between the VEGF-C dimers.

It can be speculated that the scarcity of neutralizing antibodies against VEGF-C, especially against the fully processed ΔNΔC-VEGF-C, may result from its low immunogenicity, since the amino acid sequence is extremely conserved between different species. For instance, murine ΔNΔC-VEGF-C is identical to the rat sequence and both are identical to the human sequence in 114 out of 116 amino acids (98%). In addition, the high degree of glycosylation of ΔNΔ-VEGF-C may further reduce its immunogenicity – as an example, it is known that non-glycosylated forms of therapeutic proteins like GM-CSF [36] or IFN-beta [37] greatly enhance their immunogenicity in humans.

Compared to other inhibitors of the VEGF-C – VEGF-R2/3 axis, an anti-VEGF-C antibody has the main benefit of specifically blocking the action of VEGF-C. This is important, since the VEGF/VEGF receptor interaction network is highly redundant. VEGF receptor blockers, for instance, do only block the action of a VEGF ligand on the targeted receptor, but not on other VEGF receptors activated by the ligand. At the same time, they block all interaction with the targeted receptor, indiscriminate of the VEGF ligand. Soluble VEGF-receptors (VEGF-traps) on the other hand, block all ligands that bind to them. This broad-spectrum inhibition (which might be advantageous in cancer therapy) may have impacts on other tissues, where VEGF ligand and receptor expression also occurs. For instance, VEGF-D is also expressed by osteoblasts, where it regulates bone regeneration in an autocrine manner via VEGF-R3 [14]. Interference with the VEGF-D/VEGF-R3 axis might therefore potentially affect bone regeneration. Conversely, VEGF-C is expressed in osteoclasts, where it enhances bone resorption in an autocrine manner via VEGF-R3 [38].

Taken together, we have selected a human antibody fragment that (i) binds to the receptor-binding region of mature, fully processed human VEGF-C (ΔNΔC-VEGF-C) and (ii) is capable of blocking the interaction of ΔNΔC-VEGF-C with both VEGF-R2 and VEGF-R3 at the molecular level. The V_H_ of the selected anti-VEGF-C scFvs contain camelid V_H_H-like mutations and VC2.2.2 V_H_ is sufficient to bind to ΔNΔC-VEGF-C. Upon further engineering of this minimal, 14.6 kDa antibody fragment to enhance solubility and stability, it may serve as the basis for development of bulkier fully human neutralizing anti-VEGF-C immunoproteins with improved half-lives in circulation. Such inhibitors could be useful in treatment of cancers that rely on direct VEGF-C signaling such as Kaposi sarcoma [39], acute myeloid or lymphocytic leukemia [40], [41] and cancers that metastasize via the lymphatic vasculature [42] but also against cancers that have become refractory to anti-VEGF-A treatment. Furthermore, such inhibitors could also be active against VEGF-C-induced bone degeneration and age-related macular degeneration.

## Materials and Methods

### Bacterial Media

Phage growth medium 2YT was mixed from 16 g tryptone (Fluka, Buchs, Switzerland), 10 g yeast extract (Fluka) and 5 g NaCl (Fluka) per liter. For growth of phage infected *E.coli* TG1 on solid agar plates, 2YT was supplemented with 100 µg/ml ampicillin (Sigma-Aldrich, Buchs Switzerland) and 1% (w/v) glucose (Fluka) to give 2YTAG(1%) and mixed with 17 g of agar (Hänseler, Herisau, Switzerland) per liter.

### Plasmids

The pHEN1 plasmid was used for expression of scFv in *E.coli* as described previously [19]. For expression of the V_H_ domain, the V_H_ sequence was amplified from the VC2.2.2 scFv-encoding pHEN1-based plasmid by PCR using the upstream forward primer LMB3long (attaching 5′ of the scFv coding sequence) and the downstream reverse primer VHrev (annealing at the 3′ end of V_H_) (Table 5). The PCR product was then cut with *Nco*I and *Not*I and ligated into pHEN1-backbone cut with the same enzymes. The resulting plasmid encoding VC2.2.2 V_H_ from Glu1 to Ser113 (Chothia 89–97 numbering scheme [43]) as well as the C-terminal myc-tag and amber stop codon was subsequently checked by sequencing.

**Table 5:** Primers used in this study.

Primer	Sequence (5′–3′)	Reference
LMB3long	CAGGAAACAGCTATGACCATGATTAC	[19]
fdseqlong	GACGTTAGTAAATGAATTTTCTGTATGAGG	[19]
VHrev	GAGATGAGTTTTTGTTCTGCGGCCGCactcgagacggtgaccagggt (NotI site underlined, annealing site to V_H_ in lowercase)	This study
DP47CDR1ba	TGGGTCCGCCAGGCTCCAG	[23]
DP47CDR1for	AGCCTGGCGGACCCAGCTCATMNNMNNMNNGCTAAAGGTGAATCCAGAGGCTGC	[23]
DPL16CDR1ba	TGGTACCAGCAGAAGCCAGGA	[46]
DPL16CDR1for	TCCTGGCTTCTGCTGGTACCAGCTTGCMNNMNNMNNTCTGAGGCTGTCTCCTTG	[46]
VC2.2.2heavybackmutfo	AGCCTGGCGGACCCAGCTCATATAATTCTGGCTAAAGGTGAATCCAGAGGCTGC	This study
VC2.2.3lightbackmutfo	TCCTGGCTTCTGCTGGTACCAGCTTGCAGTATTCTGTCTGAGGCTGTCTCCTTG	This study
VC2.2.13heavybackmutfo	AGCCTGGCGGACCCAGCTCATCAACGACTGGCTAAAGGTGAATCCAGAGGCTGC	This study

M  =  A/C, N = A/C/G/T.

### Antigens

Human recombinant ΔNΔC-VEGF-C was expressed from a pPICZalpha based expression vector (Invitrogen, Paisly, UK) in the yeast *Pichia pastoris* and contains an N-terminal His-tag (Figure 1). Purification to homogeneity was accomplished by IMAC affinity chromatography and gel filtration as described previously [44]. Human recombinant ΔNΔC-VEGF-C and ΔNΔC-VEGF-D, produced in mouse myeloma cells, were purchased from R&D Systems (Abingdon, UK). Human VEGF-A_165_ was kindly provided by the National Cancer Institute (Bethesda, MD). Alpha-2-macroglobulin was purchased from Sigma-Aldrich.

### Biotinylation

ΔNΔC-VEGF-C from *P. pastoris* or mammalian cells was biotinylated with SS- or LC-NHS-Biotin (Pierce, Rockford, IL). 400 µl of a 500 µg/ml protein solution in PBS (Gibco) was mixed with 80 µl of freshly prepared 1 mg/ml solution of biotinylation agent in MilliQ water and incubated for 1 hour at RT. For removal of unreacted biotinylation agent, the mixture was loaded on a PD10 gel filtration column (GE Healthcare, Glattbrugg, Switzerland), topped up with 2.12 ml PBS, eluted with 3.5 ml PBS and fractions of 0.5 ml were collected. The protein containing fractions, as measured per spectrophotometric absorbance at 280 nm, were pooled.

To check the biotinylation of the protein, 100 µl of protein solution in PBS was dotted on a nitrocellulose membrane (Biorad, Hercules, CA) with an Easy-Titer dot-blot system (Pierce). After drying for 15 min, the membrane was blocked for 1 hour with 3% BSA (Fluka) in PBS at RT. Following washing with PBS-0.1% Tween-20 (PBST), the biotinylated protein on the membrane was detected with Streptavidin-HRP (GE Healthcare) 1∶20000 in 3% BSA/PBS. The membrane was then again washed three times for 5 min with PBST, incubated with ECL (GE Healthcare) for 5 min and imaged using the GelDoc analysis system (Biorad).

### Library selections on immobilized antigen

The ETH-2-Gold antibody phage library [19] was used to pan on fully processed ΔNΔC-VEGF-C derived from *Pichia pastoris*. A Maxisorp immunotube (Nunc, Wiesbaden, Germany) was coated overnight at room temperature with 50 µ µg/ml ΔNΔC-VEGF-C in 4 ml of 100 mM carbonate buffer, pH 9.6. As control, another immunotube was incubated with carbonate buffer alone. The next morning, immunotubes were rinsed three times with PBS and blocked with 5 ml of 2% (w/v) of skimmed milk powder in PBS (MPBS) for 2 h at room temperature. After rinsing three times with PBS, ca 5×10^12^ transducing units (tu) of the phage library in 2% MPBS were added to the immunotubes. Subsequently, the immunotubes were incubated on an orbital shaker for 30 min and for another 90 min standing upright at room temperature. Unbound phage was washed away with ten washes of PBST and PBS each. For subsequent panning rounds, washing steps were increased to twenty washes with each buffer. Bound phage was eluted for 15 min by incubating the immunotubes on a rotator with 1 ml of 100 mM glycine, pH 2.2, adjusted with HCl. After collecting the eluate, the elution buffer was neutralized by adding 200 µl 1 M Tris-HCl pH 8.6. The eluted phage was subsequently used to infect 10 ml of exponentially growing *E.coli* TG1 at OD_600_ = 0.5 for 35 min at 37°C. Titration of the eluted phages, phage amplification and colony picking were performed as described previously [45].

### Affinity selections with biotinylated antigen

For selection of affinity-matured binders from the affinity maturation library, panning in solution with biotinylated antigen was used. This was done to prevent selection of binders that bind only to denatured VEGF-C. Up to 3 biopanning rounds with different concentrations of biotinylated *P. pastoris*-derived ΔNΔC-VEGF-C (VC2.1 series; 1^st^ round with 3 nM, 30 nM and 300 nM; 2^nd^ round with 30 pM and 3 nM; 3^rd^ round with 30 pM) and biotinylated mammalian cell-derived ΔNΔC-VEGF-C (VC2.2 series; 1^st^ round with 300 pM, 3 nM and 30 nM; 2^nd^ round with 3 nM) were performed. Prior to selection, 50 µl streptavidin coated magnetic beads (M-280 Dynabeads, Dynal Biotech, Oslo, Norway) per selection were blocked with 1 ml 3% BSA in PBS for 1 hour at room temperature and resuspended in 50 µl 2% BSA/PBS. 1 ml of antibody phage library (approximately 10^12^ tu in total) in 2% BSA/PBS was incubated with the biotinylated antigen at the above stated concentrations for 1 hour on a rotator at room temperature. The streptavidin magnetic beads were then added to the phage/antigen mixture and allowed to capture the biotinylated antigen and adhering phages for 15 min on a rotator at room temperature. Using a magnetic rack, the beads were subsequently washed 7 times with 1 ml each of PBS-Tween (1%) and 3 times with PBS. Then, the phages were eluted from the magnetic beads with 100 µl of 100 mM glycine, pH 2.2, adjusted with HCl, for 10 min at room temperature. The phage-containing supernatant was then removed and neutralized with 1 ml of 1 M Tris, pH 7.4. Infection of TG1 *E.coli*, titration and amplification was performed as described above.

### ELISA screening

After 2 and 3 rounds of panning, individual bacterial colonies containing the phagemid were picked and inoculated into 150 µl 2YTAG(0.1%) and grown for 3 hours in a 37°C shaking incubator. Then, the cells were induced by addition of 50 µl of 2YTA containing 4 mM isopropyl-thio-galactopyranoside (IPTG; Applichem, Darmstadt, Germany), to give a final concentration of 1 mM IPTG, and grown overnight for 30°C.

A 96-well maxisorp plate (Nunc) was coated overnight at RT with 50 µg/ml of ΔNΔC-VEGF-C in PBS as described above. The next day, the ELISA plate was washed three times with PBS and blocked with 300 µl 4% MPBS for 2 hours at RT. The plate was then washed again three times with PBS and each well was supplemented with 20 µl of 10% MPBS containing a 1∶200 dilution of mouse anti-myc 9E10 antibody (Sigma, Cat No M5546) and 1∶200 dilution of anti-mouse horseradish peroxidase labelled sheep antibody (GE Healthcare) as secondary reagents. The bacterial supernatant was centrifuged for 10 min at 1800 g and 80 µl of supernatant from each well was added to the corresponding ELISA well. The plate was then incubated for 1 hour at RT on an orbital shaker. After three washes each with PBST and PBS using a squirt bottle, 100 µl Blue-POD peroxidase substrate (Roche, Mannheim, Germany) was added to each well and the chromogenic reaction was stopped with 50 µl 1 M H_2_SO_4_ after 10 min. The plates were then read with a spectrometer at 450 nm and 650 nm. To screen for false positives, the supernatants were also tested on maxisorp plates coated with PBS alone. Since ΔNΔC-VEGF-C derived from *P. pastoris* was used for the panning, the supernatants were also tested on streptawell plates (Roche) coated with 100 µl 10^−7^ M biotinylated ΔNΔC-VEGF-C derived from mammalian cells (R&D Systems) in PBS overnight at 4°C.

### SPR analysis

All solutions to be injected into the BIAcore 3000 (GE Healthcare) were filtered using a 0.22 µm filter (Millipore, Zug, Switzerland). A streptavidin-coated sensorchip flowcell (GE Healthcare) was coated at a flowrate of 5 µl/min with 25 µl of 100 nM biotinylated ΔNΔC VEGF-C derived from either *P. pastoris* or mammalian cells in PBS, 0.01% azide, 0.005% Surfactant P-20 (GE Healthcare). For both antigens, a stable immobilization level of more than 2000 RU was achieved. Surface regeneration was done by injection of 5–10 µl 10 mM HCl. Positive supernatants from the ELISA were filtered through a 0.22 µm filter and injected as is.

### Affinity maturation

Parental antibodies obtained after 3 rounds of panning were randomized in the CDR1 at the following positions (numbering according to the Chothia 89–97 scheme [43]): 31, 32, 33 for V_H_, 31, 31a, 32 for V_L_-kappa and 31a, 31b and 32 for V_L_-lambda using degenerate primers DP47CDR1for, DPK22CDR1for and DPL16CDR1for respectively, together with DP47CDR1ba, DPK22CDR1ba, DPL16CDR1ba, LMB3long and fdseqlong respectively (Table 5). The three amplicons were assembled by PCR assembly essentially as described [19]. All primers were purchased from Sigma.

Affinity matured clones that contained a TAG amber stop codon in the mutated CDRs (coding for glutamine in suppressor strains and stop in non-suppressor strains), were backmutated to CAG by using PCR backmutation primers as specified in Table 5 and essentially the same assembly procedure as described above.

### Expression and purification of scFv antibody fragments

An overnight starter culture of 2YTAG(5%) inoculated with a single colony of phagemid bearing *E.coli* TG1 was diluted 1∶100 in 1 l of 2YTAG(0.1%) and grown at 37°C, 225 rpm until OD_600_ = 0.5. The cells were then induced by addition of IPTG (final concentration 1 mM) and grown overnight at 30°C, 225 rpm. Bacterial supernatants were clarified by centrifugation at 5000 g, 4°C for 45 min and filtered through a 0.2 µm filter (TPP, Trasadingen, Switzerland). Supernatants were then loaded on a protein-A affinity column (Biorad) using the Profinia automated protein purification system (Biorad), according to the manufacturer's recommendations. The columns were washed with Buffer A (100 mM NaCl, 0.1% Tween-20 (Sigma), 0.5 mM EDTA (Sigma) in PBS) and subsequently Buffer B (500 mM NaCl, 0.5 mM EDTA in PBS). Bound scFv were then eluted with 100 mM triethylamine (Sigma) and immediately neutralized in 1 M Tris-HCl, ph 7.0. The eluate was dialyzed overnight against PBS using Spectra/Por dialysis tubing with 12–14 kDa cutoff (Spectrum Labs, Breda, The Netherlands) and concentrated to about 1 mg/ml with Amicon Ultra 15 ultrafiltration devices with 10 kDa cutoff (Millipore). For sterilization, the scFv preparation was finally filtered through a 0.22 µm filter (Pall, Basel, Switzerland).

### SDS-PAGE and immunoblotting

Proteins were resolved on 4–12% gradient bis-tris Novex precast gels in MOPS running buffer, using LDS loading buffer with or without reducing agent as indicated by the manufacturer (all components from Invitrogen). Precision Plus molecular weight standards were from Biorad. Staining of SDS-PAGE gels was accomplished using Bio-Safe Coomassie (Biorad). For immunoblotting, the proteins resolved with SDS-PAGE were transferred to nitrocellulose membranes (Biorad). Membranes were subsequently blocked for 2 h with 5% skimmed milk powder (Coop, Basel, Switzerland) in phosphate buffered saline (PBS, Gibco) containing 0.1% (v/v) Tween-20 (Sigma), referred to as MPBST. Immunoblotting was performed using mouse anti-myc 9E10 antibody (1∶1,000, Sigma) and secondary anti-mouse HRP-labeled sheep antibody (1∶20,000, GE Healthcare), diluted in MPBST. Bands were revealed using ECL Plus detection reagent (GE Healthcare).

### Size-exclusion chromatography

Size-exclusion chromatography (SEC) and isolation of monomeric scFv was performed on an Äkta FPLC system using the Superdex 75 10/300 GL column (GE Healthcare) at a flow of 0.5 ml/min. The column was calibrated with molecular mass standards ovalbumin (43 kDa) and ribonuclease A (13.7 kDa) (GE Healthcare).

### Epitope mapping using peptide array

A peptide array (PepStar; JPT, Berlin, Germany) was used to map the epitope of the anti-VEGF-C antibody. The amino acid sequence spanning human ΔNΔC-VEGF-C from T103 to R227 and an appended C-terminal 6xHis-tag was used to define 40 overlapping, consecutive 15-mer peptides with 3 residues shift each. Synthesis of the peptides, using SPOT peptide synthesis, and peptide printing on glass slides were conducted at JPT. The myc-tag-epitope AEQLISEEDL, human and murine IgGs as well as *P. pastoris*-derived ΔNΔC-VEGF-C were printed as additional controls. Each slide features 3 identical subarrays, corresponding to 3 technical replicates per condition.

ScFvs for epitope mapping were fluorescently labelled using Cy3 and Cy5 monoreactive dye (GE Healthcare). Dye aliquots were prepared by dissolving monoreactive Cy3 or Cy5 dye, intended for labeling of 1 mg protein, in 10 µl of DMSO, dividing into 1 µl aliquots and vacuum-drying in a speed-vac. Dye aliquots were stored at 4°C in an desiccator under vacuum in the dark. 20 µl of 1 M sodium bicarbonate buffer, pH 9, was added to 200 µl of a 1 mg/ml scFv solution in PBS to bring the final pH between 8.5 and 9.5. Then, this solution was added to 2 aliquots of Cy3 or Cy5 monoreactive dye and left to incubate for 1 hour at room temperature. Non-reacted dye was subsequently quenched with 100 µl Tris buffered saline (TBS), pH 7.5. To separate free dye from the scFv-bound dye and to exchange the buffer to PBS, the solution was loaded on a microcon concentrator column YM-10 (Millipore) with a membrane-cutoff of 10 kDa. The concentrator with the scFv/dye solution was then centrifuged for three times at 14 krpm for 30 min and washed with 500 µl PBS between spins. Finally, the dye-labelled scFv was resuspended in 200 µl PBS and the protein concentration as well as the dye absorption was measured using a spectrophotometer (Nanodrop; Thermo Scientific, Wilmington, DE).

Arrays were competitively incubated essentially according to the manufacturer's instructions in a sandwich-like fashion with a Cy3-labelled anti-VEGF-C scFv and a Cy5-labelled irrelevant scFv at 30 µg/ml each, in 300 µl 0.22 µm filtered binding buffer (TBS containing 3% FBS and 0.5% Tween-20) at 4°C overnight in a humid atmosphere in the dark. Then, the microrarrays were washed 3 times for 6 min with binding buffer and 3 times for 6 min with MilliQ water in the dark. Slides were dried by spinning for 2 min at 300 rpm and scanned using a Genepix 4200A scanner (Axon Instruments, MDS Analytical Technologies, Concord, ON, Canada) with photo-multiplier tube (PMT) gain values for both channels set to prevent saturated pixels and to yield a pixel-count ratio of 1∶1 for the two channels. Scanning was done using 2 line averaging and a resolution of 5 µm. Gal-file grids were manually adjusted observing both colors and Cy3/Cy5 ratios were normalized to human and murine IgG as non-specific controls.

### VEGF-C neutralization assay using BIAcore

Unless otherwise stated, all reagents were from GE Healthcare. A CM5 sensorchip equilibrated overnight with HBS-P was activated by injection of two times 70 µl 1∶1 EDC/NHS mixture at a flow rate of 5 µl/min, resulting in a ΔRU of 214. Then, 68 µl of monoclonal anti-Fc antibody at 25 µg/ml diluted in 10 mM sodium acetate pH 5.0 immobilization buffer was injected. After 5 min, 50 µl ethanolamine was injected to deactivate the remaining binding sites. A stable immobilization level of about 16000 RU was achieved. Surface regeneration between cycles was done by injection of 5 µl of 3 M Mg_2_Cl_2_. Injections on the anti-Fc coated CM5 chip were done at a flowrate of 5 µl/min and the different proteins were diluted in HBS-P buffer. For VEGF-C/VEGF-R3 binding experiments, 100 µl of 100 nM VEGF-R3-Fc recombinant protein (R&D Systems), resulting in a response of about 2500 RU were immobilized on the anti-Fc coated CM5 chip. For VEGF-C/VEGF-R2 binding experiments, 100 µl of 100 nM VEGF-R2-Fc recombinant protein (R&D Systems), resulting in a response of about 1200 RU were immobilized on the anti-Fc coated CM5 chip.

### VEGF-C neutralization assay using competitive ELISA

A maxisorp plate was coated in quadruplicate with 100 µl of 2 µg/ml monoclonal mouse anti-human IgG (Chemicon, Temescula, CA) in PBS overnight at room temperature. The next morning, the plate was washed 3 times with PBS and blocked for 2 hours at room temperature with 200 µl blocking buffer (3% BSA (Probumin; Millipore) in PBS). The plate was then washed again 3 times with PBS and incubated with 100 µl 1 µg/ml VEGF-R2-Fc or VEGF-R3-Fc in blocking buffer for 2 hours at room temperature. After washing with 3 times PBS, 100 µl of a mixture of 2 nM biotinylated *P. pastoris*-derived ΔNΔC-VEGF-C preincubated for 30 min with a variable concentration of VC2.2.2 anti-VEGF-C scFv or irrelevant scFv in blocking buffer was added to the ELISA plate for 1 hour at room temperature. Subsequently, the ELISA plate was washed for 2 times with PBST and 1 time with PBS and a mixture of streptavidin-horseradish peroxidase conjugate diluted 1∶1000 in blocking buffer was added and incubated for 1 hour at room temperature. Finally, the plate was washed again 3 times with PBST and 3 times with PBS and developed as detailed above.
